# Parental and child-level predictors of HIV testing uptake, seropositivity and treatment initiation among children and adolescents in Cameroon

**DOI:** 10.1371/journal.pone.0230988

**Published:** 2020-04-13

**Authors:** Habakkuk A. Yumo, Rogers A. Ajeh, Isidore Sieleunou, Jackson N. Ndenkeh, Michael R. Jordan, Nadia A. Sam-Agudu, Christopher Kuaban, Thomas Loescher

**Affiliations:** 1 Research for Development International (R4D International), Yaoundé, Cameroon; 2 Center for International Health (CIH), Ludwig-Maximilians-University, Munich, Germany; 3 Faculty of Health Sciences, University of Buea, Buea, Cameroon; 4 School of Public Health, University of Montreal, Montreal, Canada; 5 Tufts University School of Medicine, Boston, Massachusetts, United States of America; 6 Institute of Human Virology Nigeria, Abuja, Nigeria; 7 Institute of Human Virology and Department of Pediatrics, University of Maryland School of Medicine, Baltimore, Maryland, United States of America; 8 Faculty of Health Sciences, University of Bamenda, Bamenda, Cameroon; Johns Hopkins Bloomberg School of Public Health, UNITED STATES

## Abstract

**Background:**

There is a growing body of evidence positioning targeted provider-initiated testing and counselling (tPITC, also known as index case testing) as a promising HIV case-finding and linkage strategy among children and adolescents. However, the effectiveness and efficiency of this strategy is limited by low HIV testing uptake and case detection rates. Despite this fact, there is very little literature on factors associated with HIV testing uptake, HIV seropositivity and ART-enrolment in tPITC implementation among African children. This study aims to bridge this information gap and contribute in improving the effectiveness and efficiency of tPITC among children and adolescents in Cameroon and beyond.

**Methods:**

In three ART clinics where tPITC was previously inexistent, we introduced the routine implementation of this strategy by inviting parents living with HIV/AIDS in care to have their biological children (6 weeks-19 years) HIV-tested. Children of consenting parents were HIV-tested; those testing positive were enrolled on ART. Parental and child-level characteristics associated with HIV testing uptake, seropositivity and ART-enrollment were assessed using bivariate and multivariate regression analysis at 5% significance level.

**Results:**

We enrolled 1,236 parents, through whom 1,990 children/adolescents were recruited for HIV testing. Among enrolled parents, 46.2% (571/1,236) had at least one child tested, and 6.8% (39/571) of these parents had at least one HIV-positive child. Among enrolled children/adolescents, 56.7% (1,129/1,990) tested for HIV and 3.5% (40/1129) tested HIV-positive. Parental predictors of HIV testing uptake among children/adolescents were sex, occupation and duration on ART: female [aOR = 1.6 (1.1–2.5)], office workers/students [aOR = 2.0 (1.2–3.3)], and parents with ART duration > 5 years [aOR = 2.0 (1.3–2.9)] had significantly higher odds to test a child than male, farmers/traders, and parents with ART duration < 5 years respectively. The only child-level predictor of testing uptake was age: children < 18 months [aOR = 5(2–10)] had significantly higher odds to test for HIV than adolescents > 15 years. Parents of children identified as HIV-positive were more likely to be female, aged 40–60 years, farmers/traders, widows/divorcees and not on ART. Children found HIV-positive and who were ART-enrolled were more likely to be female and aged 5–9 years. However, none of the above-mentioned associations was statistically significant.

**Conclusions:**

Parents who were male, farmers/traders, and on ART for ≤ 5 years were less likely to test their children for HIV. Also, adolescents 10–19 years old were less likely to be tested. Therefore, these groups should be targeted with intensive counseling and follow-up to facilitate optimal testing uptake. No association was found between parental or child-level characteristics and HIV seropositivity among tested children. This finding prompts for further research to investigate approaches to better identify and target HIV testing to children/adolescents with the highest likelihood of HIV seropositivity.

**Clinical trial registration:**

**Reg**: CinicalTrials.gov # NCT03024762.

## Introduction

Despite remarkable progress in the expansion of antiretroviral therapy (ART) coverage, children less than 15 years are still lagging behind with only 52% ART coverage compared to 59% of adults [1]. Similarly, access to and uptake of ART among adolescents 10–19 years old remains limited [2], with a global coverage of 36% [3]. This situation is more dire in West and Central African countries like Cameroon, where pediatric ART coverage is 25% compared to 51% in adults [1]. In West and Central Africa, regional pediatric ART coverage is estimated at 26%, which is less than half of the 59% pediatric ART coverage in the East and Southern African Region [1].

Poorly-implemented and/or ineffective case-finding and linkage to care strategies are major factors contributing to the sub-optimal performance of pediatric and adolescent ART programs [4–6]. However, there is a growing body of evidence positioning index case testing as a promising case-finding and linkage strategy in this sub-population. Already described in the early 2000s [7], index case testing among children is grounded in the knowledge that > 90% of pediatric HIV infections result from vertical transmission of HIV [8]. Thus, targeting for HIV testing children of parents known to be living with HIV (index cases) is a potentially high-yield case finding and linkage approach, especially when compared to universal provider-initiated testing and counselling [9]. Index case testing for children was first recommended by UNICEF and WHO in 2010 [10] with variable levels of implementation across countries [11]. Recent operational research from Kenya, Malawi and Cameroon have shown that index case testing is acceptable, feasible and effective in HIV case-identification and timely ART enrollment for children and adolescents [9,12–14]. In a previous publication, we named this strategy, “targeted provider initiated testing and counselling (tPITC)” as opposed to the universal or blanket PITC (bPITC) [9]. In that article, we demonstrated the superiority of tPITC over the bPITC in terms of case detection and ART enrollment among Cameroonian children and adolescents. We reported that although tPITC acceptability among parents was extremely high at 99.7%, HIV testing uptake was moderate at 56.7% and ART linkage was 85% [9]. In Kenya, Wagner et al. found that among 48% of in-care parents living with HIV who consented to testing their eligible children for HIV, only 14% ultimately tested one or more children [12]. In Malawi, Ahmed et al. reported a consent for testing and linkage rate of 94% and 77% respectively, among children/adolescents in the context of tPITC implementation [13]. This available evidence suggests that with improved testing uptake and linkage rates, tPITC will potentially be more effective and efficient, and contribute to closing the current gap in pediatric and adolescent ART coverage. There is however still limited knowledge on factors influencing HIV testing uptake, seropositivity and ART-enrollment among children and adolescents within the context of tPITC. Such data is needed to better target children/adolescents with higher likelihood of HIV seropositivity, especially in relatively low-prevalence, high-burden, limited-resource settings. This study investigated for parental and child-related predictors of testing uptake, seropositivity and treatment initiation during tPITC implementation among children and adolescents in Cameroon.

## Materials and methods

### Study design and setting

This article is drawn from a sub-analysis of the dataset for “Active Search for Pediatric HIV/AIDS” (ASPA), a cross-sectional study assessing the acceptability, feasibility and effectiveness of tPITC versus bPITC in Cameroon [9]. The ASPA study was implemented at three government hospitals: Limbe Regional Hospital (LRH) in the South-West, Ndop District Hospital (NDH) in the North-West and Abong-Mbang District Hospital (ADH) in the Eastern Region. These facilities provide comprehensive healthcare services, including HIV prevention and treatment and were purposefully selected for inclusion of urban, semi-urban and rural populations. Prior to this study, there was no tPITC implementation in the aforementioned hospitals. This intervention was newly-introduced as routine care in these health facilities during the study period.

### Study period and population

The study was implemented at LRH from July through December 2015, and from June through November 2016 at the two other sites. The study population was adults living with HIV in care at the ART clinics and their biological children of unknown HIV status aged 6 weeks to 19 years old. Critically ill parents or children, and children with known HIV-positive status were excluded from the study.

### Study procedures

#### Site preparation

This included staff training on tPITC implementation, provision of HIV testing kits, data collection and monitoring tools, and additional human resources to support study implementation. Specifically, study staff were recruited to work collaboratively with hospital staff to support ASPA implementation and ensure compliance of the study protocol.

#### Participant enrolment

Current patients visiting ART clinics for drug refills or new patients referred for enrollment in HIV care were approached by a trained counsellor for study recruitment. Consenting eligible parents were invited to enroll their biological children in the study for HIV testing, either in the hospital or at home/community.

#### HIV testing, linkage and ART enrolment

For children <18 months old, blood specimens were collected on filter paper and shipped to reference laboratories for HIV DNA PCR testing. For children ≥18 months old, HIV testing was conducted using two rapid tests. Rapid HIV testing was conducted per the national HIV testing algorithm [15] by trained laboratory technicians at the study hospitals or in the community by trained community health workers. Pre and post-test counselling, including result release to parents/children was conducted according to national guidelines on HIV management [15]. Children/adolescents who had previously tested for HIV and had a negative or unknown HIV result were re-tested.

The WHO test and treat policy [16] was not established at study sites at study initiation, however implementation was subsequently scaled up. Thus, in some cases, children testing HIV-positive were assessed for ART eligibility using the WHO clinical staging and/or baseline laboratory analysis including CD4 count. Eligible children were initiated on ART following national guidelines [15], which was adapted from WHO’s 2013 HIV prevention and management guidelines [17]. In other cases, children testing HIV-positive were enrolled on ART following WHO’s test and treat policy [16].

### Data collection, management, analysis and outcome measurement

Data on tPITC implementation and outcomes were collected prospectively during the study period. For this purpose, we designed a specific study register for the identification and enumeration of children of HIV-infected parents in care at the ART clinic. In this register, we tracked each parent’s ART clinic code, the number of eligible biological children, and the HIV testing appointment date for the child. During ART drug refills (current patients) or enrolment visits (new patients), a pre-tested structured questionnaire was used by a trained data clerk to collect socio-demographic information and HIV history of both parents and children. In addition, for each child presented for HIV testing, we completed a follow-up form capturing HIV test results, plus clinical assessment data for those HIV-positive. De-identified data were entered into a Microsoft Access database and analyzed using Stata 2015 (StataCorp, LLC, Texas, USA).

The study outcomes were parental and child-level factors associated with HIV testing uptake, HIV seropositivity and ART-enrollment among children and adolescents. Depending on the situation, these outcomes were assessed using Pearson chi-square/Fisher's exact test or bivariate and multivariate logistic regression at 5% significance level.

### Ethical considerations

Participation in the study was voluntary for both parents and children. Only parents who consented were enrolled and assent was requested from children above 11 years of age. Written informed consent was obtained from participating parents. Likewise, assent for children over the age of 11 was obtained through a signed written assent form. The ASPA study received ethical approval from the Cameroon National Ethics Committee, the Ludwig-Maximilians-Universität, Munich (Germany), and the Albert Einstein College of Medicine (NY, U.S.) [9].

## Results

### Parental predictors of HIV testing uptake, seropositivity and ART-enrolment among children and adolescents

A total of 1,240 eligible parents were counselled and enrolled for HIV testing for their children. Of these, 1,236 (99.7%) consented to test their children and they were enrolled in the study [9]. The majority of parents were females (80.3%) and on ART (94.9%). Among the 1,236 consenting parents, only 46.2% (571/1236) ultimately tested at least one child. The main reasons for failure to test children were lack of transport fare and limited time to bring children to the hospital, and children not living in the same household with their parents [9]. In bivariate analysis, associations were observed between testing at least one child and the following parental covariates: sex, age, educational level, marital status, occupation and duration on ART. Controlling for confounding variables in multivariate logistic regression, males were 40% less likely to test at least one child compared to females [aOR = 0.6 ((0.4–0.9); p = 0.012]; office workers/students were almost 2 times more likely to test at least one child compared to farmers/traders [aOR = 2.0 (1.2–3.3); p = 0.014]; singles were 20% less likely to test at least one child compared to married/cohabitating parents (aOR = 0.8 (0.6–1.1); p = 0.006); parents who had been on ART for more than 5 years were twice as likely to test at least one child compared to those who had been on ART for 5 years or less (aOR = 2.0 (1.3–2.9; p<0.001) (Table 1).

**Table 1:** Parental characteristics and HIV testing uptake in biological children in three hospitals, Cameroon.

*Characteristics*	Total parents enrolled (N = 1236)	Parents who tested at least 1 child (N = 571)	Bivariate Logistic Regression		Multivariate Logistic Regression	
	n (%)	n (%)	OR (95% CI)	p	OR (95% CI)	p
***Sex***				0.069		0.012
*Female (Ref)*	992 (80.3)	471 (80.2)				
*Male*	244 (19.7)	100 (17.5)	0.7 (0.5–1.0)		0.6 (0.4–0.9)	
***Age (years)***				0.02		0.657
*0–24 (Ref)*	73 (5.9)	39 (53.4)				
*25–39*	718 (58.1)	349 (48.6)	0.8 (0.5–1.3)		0.9 (0.5–1.5)	
*40–60*	445 (36.0)	183 (41.1)	0.6 (0.3–1.0)		0.8 (0.4–1.4)	
***Education level***				0.005		0.195
*None (Ref)*	45 (3.6)	12 (26.6)				
*Primary*	709 (57.4)	316 (44.5)	2.2 (1.1–4.3)		1.7 (0.8–3.6)	
*Secondary/high school*	482 (39.0)	243 (50.4)	2.7 (1.4–5.5)		1.9 (0.9–4.0)	
***Occupation***				<0.001		0.014
*Farming/trading (Ref)*	859 (69.5)	367 (42.7)				
*Office work/student*	95 (7.6)	58 (61.0)	1.4 (1.1–1.9)		2.0 (1.2–3.3)	
*Others*	282 (22.8)	146 (51.7)	2.1 (1.3–3.2)		1.4 (0.9–1.7)	
***Marital status***				0.001		0.006
*Married/Cohabitating (Ref)*	660 (53.4)	327 (49.5)				
*Single*	261 (21.1)	128 (49.0)	0.9 (0.7–1.3)		0.8 (0.6–1.1)	
*Widow/Divorced*	315 (25.5)	116 (36.8)	0.5 (0.4–0.7)		0.6 (0.4–0.8)	
***Currently on ART***				0.453		
*No (Ref)*	63 (5.1)	32 (50.7)				
*Yes*	1173 (94.9)	539 (45.9)	0.8 (0.5–1.4)			
***Duration on ART (years)***				<0.001		<0.001
*≤5 (Ref)*	1 014 (88.1)	449 (44.3)				
*>5*	137 (11.9	87 (63.5)	2.1 (1.5–3.1)		2.0 (1.3–2.9)	

Among the 571 parents who enrolled in the study and tested at least 1 child, 39 (6.8%) were found with at least 1 HIV-positive child. Of those, 34 (87.2%) enrolled at least one child on ART. Parents with children diagnosed HIV-positive were more likely females, aged 40–60 years, farmers/traders, widows/divorcees and not on ART. On the other hand, parents who enrolled HIV-positive children on ART were predominantly female (85.3%), aged 25–39 years (50.0%), farmers/traders (76.5%), married/cohabitating (50.0%) and on ART (88.2%). However, none of the above-mentioned associations was statistically significant (Table 2).

**Table 2:** Characteristics of parents found with HIV positive and who enrolled children on ART at three hospitals, Cameroon.

*Characteristics*	Parents who tested at least one child (N = 571)	Parents with at least one HIV+ child (N = 39)	Bivariate Logistic Regression-parents who tested at least one child (N = 571)		Parental characteristics and ART enrollment for children (N = 34)*	
	N (column%)	n (row%)	OR (95% CI)	P	n (row%)	P
***Sex***				0.717		0.999
*Female (Ref)*	471 (82.5)	33 (7.0)			29 (87.9)	
*Male*	100 (17.5)	6 (6.0)	0.8 (0.3–2.1)		5 (83.3)	
***Age (years)***				0.282		0.82
*0–24 (Ref)*	39 (6.8)	2 (5.1)			2 (100.0)	
*25–39*	349 (61.1)	20 (5.7)	1.1 (0.3–5.0)	0.877	17(85.0)	
*40–60*	183 (32.0)	17 (9.3)	1.9 (0.4–8.6)	0.406	15 (88.2)	
***Occupation***				0.484		0.106
*Farming/trading (Ref)*	367 (64.3)	28 (7.6)			26 (92.9)	
*Office work/student*	58 (10.2)	2 (3.4)	0.4 (0.1–1.9)	0.261	2 (100.0)	
*Others*	146 (25.6)	9 (6.2)	0.8 (0.4–1.8)	0.563	6 (66.7)	
***Civil status***				0.215		0.108
*Married/cohabitating (Ref)*	327 (57.3)	18 (5.5)			17 (94.4)	
*Single*	128 (22.4)	9 (7.0)	1.3 (0.6–3.0)	0.536	6 (66.7)	
*Widow/Divorced*	116 (20.3)	12 (10.3)	2.0 (0.9–4.3)	0.079	11 (91.7)	
***Currently on ART***				0.05		0.517
*No (Ref)*	32 (5.6)	5 (15.6)			4 (80.0)	
*Yes*	539 (94.4)	34 (6.3)	0.4 (0.1–1.0)	0.05	30 (88.2)	

*Pearson Chi-Square or Fisher’s Exact test

### Child-level predictors of HIV testing uptake, seropositivity and ART-enrolment among children and adolescents

Altogether, parents in HIV care at the three hospitals reported a total of 3,195 children/adolescents; and among these 1,990 (62.3%) were eligible for HIV testing. Approximately half (50.2%) of children/adolescents were females and predominantly (31.8%) between 5 and 9 years of age.

Among the 1,990 children enrolled, only 34.7% (691/1990) had ever been previously tested for HIV. Of these 691 children, 98.4% (680) and 1.6% (11) reported having negative and unknown test results, respectively. Among the 1,990 children enrolled, 56.7% (1,129) underwent HIV testing during the study. Most HIV testing was performed in the hospital (82.5%, 931/1129) with only 17.5% (198/1129) of parents opting for home-based HIV testing of their children. In bivariate analysis, child-level covariates associated with HIV testing uptake were: age and educational level. Controlling for confounders in multivariate logistic regression, age was the only independent predictor for HIV testing uptake among children/adolescents (Table 3).

**Table 3:** Children/adolescents characteristics and HIV testing uptake at three hospitals, Cameroon.

*Characteristics*	Total Children (N = 1990)	Children who tested for HIV in each category (N = 1129)	Bivariate Logistic Regression		Multivariate Logistic Regression	
	n (%)	n (%)	OR (CI)	P	OR (CI)	P
***Sex***				0.355		
*Female (Ref)*	999 (50.2)	577 (57.7)				
*Male*	991 (49.8)	552 (55.7)	0.9 (0.7–1.0)			
***Age***				<0.001		<0.001
*0–17 months (Ref)*	162 (8.1)	132 (81.5)				
*18–59 months*	390 (19.6)	244 (62.5)	0.3 (0.2–0.5)		0.3 (0.2–0.6)	
*5–9 years*	632 (31.8)	369 (58.3)	0.3 (0.2–0.4)		0.3 (0.2–0.6)	
*10–14 years*	520 (26.1)	256 (49.2)	0.2 (0.1–0.3)		0.2 (0.1–0.4)	
*15–19 years*	286 (14.4)	120 (44.7)	0.2 (0.1–0.3)		0.2 (0.1–0.5)	
***Education level***				<0.001		0.076
*None (Ref)*	424 (21.3)	296 (69.8)				
*Primary*	1058 (53.2)	606 (57.2)	0.5 (0.4–0.7)		0.8 (0.6–1.2)	
*Secondary/high school*	508 (25.5)	227 (44.6)	0.3 (0.2–0.4)		0.6 (0.4–1.0)	

Of the 1129 children/adolescents who tested for HIV, 40 (3.5%) were found HIV-positive. Among children/adolescents diagnosed HIV-positive, 35 (87.5%) were ART-enrolled. Children found HIV positive and those enrolled on ART were more likely to be female, aged 5–9 years and at primary school educational level. However, none of these associations was statistically significant (Table 4).

**Table 4:** Children-level characteristics and HIV-seropositivity at three hospitals, Cameroon.

*Characteristics*	Children who tested for HIV (N = 1129)	Children tested HIV+ (N = 40)	Bivariate logistic Regression- children who tested for HIV (N = 1129)		Children-level characteristics and ART enrollment (N = 35)***	
	N (column%)	n (row%)	OR (95% CI)	p	n (row%)	P
***Sex***				0.638		0.155
*Female (Ref)*	551 (51.4)	22 (4.0)			21 (95.5)	
*Male*	522 (48.6)	18 (3.4)	0.9 (0.5–1.6)		14 (77.8)	
***Age***				0.181		0.117
0-17m*(Ref)	80 (7.5)	3 (3.8)			3 (100.0)	
*18-59m*	244 (22.7)	6 (2.5)	0.6 (0.2–2.6)		6 (100.0)	
5–9 y**	367 (34.2)	20 (5.4)	1.5 (0.4–5.1)		18 (90.0)	
*10–14 y*	254 (23.7)	5 (2.0)	0.5 (0.1–2.2)		5 (100.0)	
*15–19 y*	128 (11.9)	6 (4.7)	1.3 (0.3–5.2)		3 (50.0)	
***Educational level***				0.857		0.091
*None (Ref)*	247 (23.0)	8 (3.2)			8 (100.0)	
*primary*	600 (55.9)	24 (4.0)	1.2 (0.6–2.8)		22 (91.7)	
*Secondary/high school*	226 (21.1)	8 (3.5)	1.1 (0.4–3.0)		5 (62.5)	

m* = age in months; y** = age in years,

***Pearson Chi-Square or Fisher’s Exact test

## Discussion

Previous studies have reported on the acceptability, utilization and effectiveness of tPITC in sub-Saharan African countries [9,12–14]. However, little was known on parental and child-level characteristics affecting the outcome of this strategy. This study indicates that tPITC outcomes can be influenced by both parental and child-level characteristics.

The large majority (80.2%) of parents who tested children was female. This is in line with the feminization of the HIV services uptake and the HIV epidemic in Cameroon and in other sub-Saharan African countries [18, 19]. Mothers were 60% more likely to test at least one child, compared to fathers (aOR = 0.6 (0.4–0.9); p = 0.012). Structured interventions targeting parents in HIV care, especially men could improve HIV testing uptake among children/adolescents. For example, non-financial and financial incentives proven to be effective in increasing uptake of HIV services in other populations could be evaluated in tPITC [20, 21].

The vast majority of parents (93.6%) preferred facility-based HIV testing for their children. This is in sharp contrast to a study in Kenya where 88% of index parents preferred home-based HIV testing for their children [13]. This difference could be due to the lower HIV stigma index in Kenya (40.0%) [22] compared to Cameroon (68.7%) [23] and suggest that less HIV stigmatizing communities may be more friendly to the uptake of community HIV services.

Parents with secondary/high school education level were more likely to test at least one child (OR = 2.7 (1.4–5.5); p = 0.005) compared to those with no formal education. Though this difference was not statistically significant after controlling for confounders (aOR = 1.9 (0.9–4.0) p = 0.195), it opposed the findings of studies conducted before 1999 in low- and middle-income countries (including Cameroon), showing lower uptake of HIV services among the most educated populations [24]. Rather, this finding is consistent with studies conducted after 1999 showing a positive association between educational attainment and the uptake of HIV services in Sub-Saharan Africa [25–27].

Compared to parents who have been on ART for ≤ 5 years, those with ART duration > 5 years were twice as likely to test at least one child (aOR = 2.0 (1.3–2.9); p<0.001). This is in line with increased HIV/AIDS awareness (including pediatric HIV services) of parents in HIV care over time.

In a previous article we reported that tPITC had a higher yield compared to the universal HIV testing (bPITC) approach. Actually, the number of parents needed to counsel to detect a new HIV infected child/adolescent was 31 for tPITC against 62 for bPITC [9]. However, this study shows that out of 1,236 parents enrolled in the study and who tested at least one child, 39 (3.2%) were found with at least one HIV-positive child. This indicates the need for a more targeted approach in tPITC (index case testing) so as to enroll HIV-positive parents with a higher likelihood of having an HIV-positive child. Actually, there was a non-statistically significant association between HIV-seropositivity in children and female parents, aged 40–60 years, farmers/traders, widows/divorcees, and not on ART (S2 Table). Further research examining these factors could guide more efficient strategies for tPITC implementation.

Only 34.7% of children in our cohort had ever been tested for HIV before the study. This is consistent with the 33.4% of children reporting history of HIV testing in an index case HIV testing in Kenya [13]. It also aligns with the 36.5% of adolescents and young people aged 15–24 years reporting a history of HIV testing in a population-based survey in sub-Saharan Africa [28]. Despite adequate knowledge, coupled with the availability of effective diagnostic technology as well as apparent parental willingness to have their children tested for HIV [29], it appears that HIV programs still fail to reach and optimize HIV testing and treatment coverage for children/adolescents. These missed opportunities are unacceptable and require urgent remedies to translate available knowledge and technology into practice.

Comparing the reference age category (0–17 months) with the other age categories, we found that the higher the child’s age, the lower the likelihood for them to be tested for HIV. For example, children <18 months old were 5 times more likely to test for HIV compared to adolescents aged 15–19 years (aOR = 0.2 (0.1–0.5; p<0.001) (S3 Table). Older children and adolescents may not be readily available to go the hospital for HIV testing, especially when are asymptomatic. We reported in a previous article (from the same dataset) that school attendance was a reason of failure to test a child during tPITC implementation [9]. This is consistent with the finding of this study showing that children/adolescents of secondary/high school education level were 40% less likely to test for HIV compared to those with no/primary education (S3 Table). Thus, tPITC implementers could consider other means of reaching out to older children/adolescents with HIV testing. For example, community-based HIV testing including school-based programs among children and adolescents had shown to be effective in HIV case detection in several African countries [30–32]. The potential of these outreach interventions in optimizing tPITC outcomes could be further explored.

The majority of newly HIV diagnosed children (72.5%) as well as those initiated on ART (77.1%) were children <10 years old (S4 Table). It is established that AIDS-related mortality is highest among neonates, infants and children not receiving ART [33–34]. Therefore, if well-implemented, tPITC could help reduced mortality related to untreated HIV infection. In the same vein, the large majority of newly-diagnosed children (85.0%) and those initiated on ART (91.2%) were children less than 15 years old (S4 Table). This was not because the yield in this category was higher (S4 Table), but because the large majority of tested children were less than 15 (S3 Table). The age category “children less than 15 years” is the age disaggregate for the UNAIDS global AIDS monitoring indicators for pediatric HIV treatment [35]. Though the yield in this category was not higher, the finding suggests that if implemented at large scale, tPITC could contribute in achieving the UNAIDS 95-95-95 targets in children and adolescents by 2030 [36].

With only 40 (3.5%) children/adolescents diagnosed HIV-positive out of 1,129 HIV-tested, there is also a need to better target children/adolescents with a higher likelihood of HIV-positivity in tPITC implementation. Further investigations are required to elucidate better approaches to improve the yield of tPITC. A outpatient screening tool for HIV testing among older children and adolescents has been shown effective in increasing yield for HIV testing in Zimbabwe [37]. However, this tool was validated in a higher HIV prevalence settings in Zimbabwe, and among children/adolescents who did not necessarily have an index parent living with HIV [37]. This still leaves a relatively unexplored gap for predictors of HIV testing uptake and positivity yield for tPITC especially in low prevalence settings.

### Strengths and limitations

The strength of our study lies in our evaluation of patient and parent-level characteristics that may influence HIV testing uptake, positivity and ART enrollment for infants, children and adolescents across a wide age range (6 weeks-19 years). This new knowledge could guide policy-makers and other stakeholders [38,39] in the design and implementation of more effective case-identification in settings such as Cameroon with relatively low HIV prevalence. The study is limited by the fact that the sites were not randomly selected and did not include the Northern regions of Cameroon, and thus results may not be generalizable nationwide. That notwithstanding, the external validity of our study is stronger as it was conducted in 3 hospitals from three different geographic locations covering urban, semi-urban and rural populations in Cameroon. Also, the relatively small sample size, may have limited our ability to conduct robust analysis for parental and child-level predictors of HIV-positivity and ART-enrollment. However, our findings have provided preliminary data that will guide further research in this area.

## Conclusions and recommendations

In resource-limited, high-burden settings, targeted HIV case identification strategies are critically important to maximize efficiency and effectiveness. Our findings suggest that even under the potentially high-yield index case testing strategy, further differentiated approaches may be needed to bridge the gap between parental/child acceptance, successfully testing the child/adolescent, and ultimately, linkage to treatment. Further quantitative and qualitative studies are needed to identify robust predictors of uptake, yield and treatment initiation in order to optimize case finding and treatment among children and adolescents within the context of tPITC implementation in Cameroon and similar HIV low-prevalence, high-stigma settings.

## Supporting information

S1 Table Parental characteristics and HIV testing uptake among biological children in three hospitals, Cameroon. (DOCX)

S2 Table Parental characteristics, HIV seropositivity and ART-enrollment among biological children at three hospitals, Cameroon. (DOCX)

S3 Table Child/adolescent characteristics and HIV testing uptake at three hospitals, Cameroon. (DOCX)

S4 Table Child/adolescent characteristics and HIV-seropositivity at three hospitals, Cameroon. (DOCX)

S1 Dataset Parents. (XLSX)

S2 Dataset Children. (XLS)
