# Supplementary material for: Parental and child-level predictors of HIV testing uptake, seropositivity and treatment initiation among children and adolescents in Cameroon
Source: PLoS One. 2020 Apr 13;15(4):e0230988. doi: 10.1371/journal.pone.0230988 (PMC7153850; doi:10.1371/journal.pone.0230988)
Supplement: S1 Table — (DOCX) [file pone.0230988.s001.docx]

| **Table 1: Parental characteristics and HIV testing uptake in biological children in three hospitals, Cameroon** | | | | | | |
| --- | --- | --- | --- | --- | --- | --- |
| **Characteristics** | **Total Parents (N=1236)** | **Parents who tested at least one child (N= 571)** | **Bivariate Logistic Regression** | | **Multivariate Logistic Regression** | |
|  | **n (column%)** | **n (row%)** | **OR (95% CI)** | **p** | **OR (95% CI)** | **p** |
| **Sex** |  |  |  | 0.069 |  | 0.012 |
| Female (Ref) | 992 (80.3) | 471 (47.5) |  |  |  |  |
| Male | 244 (19.7) | 100 (41.0) | 0.8 (0.6-1.0) |  | 0.6 (0.5-0.9) |  |
| **Age group (years)** |  |  |  | 0.02 |  | 0.657 |
| 0-24 (Ref) | 73 (5.9) | 39 (53.4) |  |  |  |  |
| 25-39 | 718 (58.1) | 349 (48.6) | 0.8 (0.5-1.3) |  | 1.0 (0.6-1.7) |  |
| 40-60 | 445 (36.0) | 183 (41.1) | 0.6 (0.4-1.0) |  | 0.9 (0.5-1.6) |  |
| **Education level** |  |  |  | 0.005 |  | 0.195 |
| None (Ref) | 45 (3.6) | 12 (26.7) |  |  |  |  |
| Primary | 709 (57.4) | 316 (44.6) | 2.2 (1.1-4.3) |  | 1.9 (0.9-3.8) |  |
| Secondary/high school | 482 (39.0) | 243 (50.4) | 2.8 (1.4-5.5) |  | 2.0 (0.9-4.1 ) |  |
| **Occupation** |  |  |  | <0.001 |  | 0.014 |
| Farming/trading (Ref) | 859 (69.5) | 367 (42.7) |  |  |  |  |
| Office work/student | 95 (7.7) | 58 (61.1) | 2.1 (1.4-3.2) |  | 2.0 (1.2- 3.3) |  |
| Others | 282 (22.8) | 146 (51.8) | 1.4 (1.1-1.9) |  | 1.3 (0.9-1.7) |  |
| **Civil status** |  |  |  | 0.001 |  | 0.006 |
| Married/Cohabitating (Ref) | 660 (53.4) | 327 (49.5) |  |  |  |  |
| Single | 261 (21.1) | 128 (49.0) | 1.0 (0.7-1.3) |  | 0.8 (0.6-1.1) |  |
| Widow/Divorced | 315 (25.5) | 116 (36.8) | 0.6 (0.4-0.8) |  | 0.6 (0.4-0.8) |  |
| **Currently on ART** |  |  |  | 0.453 |  |  |
| No (Ref) | 63 (5.1) | 32 (50.8) |  |  |  |  |
| Yes | 1173 (94.9) | 539 (46.0) | 0.8 (0.5-1.4) |  |  |  |
| **Duration on ART (years)** |  |  |  | <0.001 |  | <0.001 |
| ≤5 (Ref) | 1014 (88.1) | 449 (44.3) |  |  |  |  |
| >5 | 137 (11.9) | 87 (63.5) | 2.2 (1.5-3.2) |  | 2.0 (1.4-3.0) |  |
