# Supplementary material for: Parental and child-level predictors of HIV testing uptake, seropositivity and treatment initiation among children and adolescents in Cameroon
Source: PLoS One. 2020 Apr 13;15(4):e0230988. doi: 10.1371/journal.pone.0230988 (PMC7153850; doi:10.1371/journal.pone.0230988)
Supplement: S2 Table — (DOCX) [file pone.0230988.s002.docx]

| **Table 2: Characteristics of parents found with HIV positive and who enrolled**  **Children on ART at three hospitals, Cameroon** | | | | | | |
| --- | --- | --- | --- | --- | --- | --- |
| **Characteristics** | **Parents who tested at least one child (N=571)** | **Parents with at least one HIV+ child (N=39)** | **Bivariate Logistic Regression-parents who tested at least one child (N=571)** | | **Parental characteristics and ART enrollment for children (N=34)*** | |
|  | **N (column%)** | **n (row%)** | **OR (95% CI)** | **p** | **n (row%)** | **P** |
| **Sex** |  |  |  | 0.717 |  | 0.999 |
| Female (Ref) | 471 (82.5) | 33 (7.0) |  |  | 29 (87.9) |  |
| Male | 100 (17.5) | 6 (6.0) | 0.8 (0.3-2.1) | 0.717 | 5 (83.3) |  |
| **Age (years)** |  |  |  | 0.282 |  | 0.82 |
| 0-24 (Ref) | 39 (6.8) | 2 (5.1) |  |  | 2 (100.0) |  |
| 25-39 | 349 (61.1) | 20 (5.7) | 1.1 (0.3-5.0) | 0.877 | 17(85.0) |  |
| 40-60 | 183 (32.0) | 17 (9.3) | 1.9 (0.4-8.6) | 0.406 | 15 (88.2) |  |
| **Occupation** |  |  |  | 0.484 |  | 0.106 |
| Farming/trading (Ref) | 367 (64.3) | 28 (7.6) |  |  | 26 (92.9) |  |
| Office work/student | 58 (10.2) | 2 (3.4) | 0.4 (0.1-1.9) | 0.261 | 2 (100.0) |  |
| Others | 146 (25.6) | 9 (6.2) | 0.8 (0.4-1.8) | 0.563 | 6 (66.7) |  |
| **Civil status** |  |  |  | 0.215 |  | 0.108 |
| Married/cohabitating (Ref) | 327 (57.3) | 18 (5.5) |  |  | 17 (94.4) |  |
| Single | 128 (22.4) | 9 (7.0) | 1.3 (0.6-3.0) | 0.536 | 6 (66.7) |  |
| Widow/Divorced | 116 (20.3) | 12 (10.3) | 2.0 (0.9-4.3) | 0.079 | 11 (91.7) |  |
| **Currently on ART** |  |  |  | 0.05 |  | 0.517 |
| No (Ref) | 32 (5.6) | 5 (15.6) |  |  | 4 (80.0) |  |
| Yes | 539 (94.4) | 34 (6.3) | 0.4 (0.1-1.0) | 0.05 | 30 (88.2) |  |
| P*= Pearson-Square or Fisher's Exact test | | | | | | |
