# Supplementary material for: Parental and child-level predictors of HIV testing uptake, seropositivity and treatment initiation among children and adolescents in Cameroon
Source: PLoS One. 2020 Apr 13;15(4):e0230988. doi: 10.1371/journal.pone.0230988 (PMC7153850; doi:10.1371/journal.pone.0230988)
Supplement: S3 Table — (DOCX) [file pone.0230988.s003.docx]

| **Table 3: Children/adolescents characteristics and HIV testing uptake at three hospitals, Cameroon** | | | | | | |
| --- | --- | --- | --- | --- | --- | --- |
| **Characteristics** | **Total Children (N=1990)** | **Children who tested for HIV (N=1129)** | **Bivariate Logistic Regression** | | **Multivariate Logistic Regression** | |
|  | **n (column%)** | **n (row%)** | **OR (95% CI)** | **p** | **OR (95% CI)** | **p** |
| **Sex** |  |  |  | 0.355 |  |  |
| Female (Ref) | 999 (50.2) | 577 (57.8) |  |  |  |  |
| Male | 991 (49.8) | 552 (55.7) | 0.9 (0.8-1.1) |  |  |  |
| **Age** |  |  |  | <0.001 |  | <0.001 |
| 0-17 months (Ref) | 162 (8.1) | 132 (81.5) |  |  |  |  |
| 18-59 months | 390 (19.6) | 244 (62.6) | 0.4(0.2-0.6) |  | 0.4 (0.2-0.6) |  |
| 5-9 years | 632 (31.8) | 369 (58.4) | 0.3 (0.2-0.5) |  | 0.4 (0.2-0.6) |  |
| 10-14 years | 520 (26.1) | 256 (49.2) | 0.2 (0.1-0.3) |  | 0.3 (0.2-0.5) |  |
| 15-19 years | 286 (14.4) | 128 (44.8) | 0.2 (0.1-0.3) |  | 0.3 (0.1-0.5) |  |
| **Education level** |  |  |  | <0.001 |  | 0.076 |
| None (Ref) | 424 (21.3) | 296 (69.8) |  |  |  |  |
| Primary | 1058 (53.2) | 606 (57.3) | 0.6 (0.5-0.7) |  | 0.9 (0.6-1.3) |  |
| Secondary/high school | 508 (25.5) | 227 (44.7) | 0.3 (0.3-0.5) |  | 0.6 (0.4-1.0) |  |
