# Supplementary material for: Parental and child-level predictors of HIV testing uptake, seropositivity and treatment initiation among children and adolescents in Cameroon
Source: PLoS One. 2020 Apr 13;15(4):e0230988. doi: 10.1371/journal.pone.0230988 (PMC7153850; doi:10.1371/journal.pone.0230988)
Supplement: S4 Table — (DOCX) [file pone.0230988.s004.docx]

| **Table 4: Children-level characteristics and HIV-seropositivity at three hospitals, Cameroon** | | | | | | |
| --- | --- | --- | --- | --- | --- | --- |
| **Characteristics** | **Children who tested for HIV (N=1129)** | **Children tested HIV+ (N=40)** | **Bivariate logistic Regression- children who tested for HIV (N=1129)** | | **Children-level characteristics and ART enrollment (N=35)***** | |
|  | **N (column%)** | **n (row%)** | **OR (95% CI)** | **p** | **n (row%)** | **P** |
| **Sex** |  |  |  | 0.638 |  | 0.155 |
| Female (Ref) | 551 (51.4) | 22 (4.0) |  |  | 21 (95.5) |  |
| Male | 522 (48.6) | 18 (3.4) | 0.9 (0.5-1.6) | 0.638 | 14 (77.8) |  |
| **Age** |  |  |  | 0.181 |  | 0.117 |
| 0-17m*(Ref) | 80 (7.5) | 3 (3.8) |  |  | 3 (100.0) |  |
| 18-59m | 244 (22.7) | 6 (2.5) | 0.6 (0.2-2.6) | 0.545 | 6 (100.0) |  |
| 5-9 y** | 367 (34.2) | 20 (5.4) | 1.5 (0.4-5.1) | 0.535 | 18 (90.0) |  |
| 10-14 y | 254 (23.7) | 5 (2.0) | 0.5 (0.1-2.2) | 0.372 | 5 (100.0) |  |
| 15-19 y | 128 (11.9) | 6 (4.7) | 1.3 (0.3-5.2) | 0.747 | 3 (50.0) |  |
| **Educational level** |  |  |  | 0.857 |  | 0.091 |
| None (Ref) | 247 (23.0) | 8 (3.2) |  |  | 8 (100.0) |  |
| primary | 600 (55.9) | 24 (4.0) | 1.2 (0.6-2.8) | 0.598 | 22 (91.7) |  |
| Secondary/high school | 226 (21.1) | 8 (3.5) | 1.1 (0.4-3.0) | 0.857 | 5 (62.5) |  |
| m*= age in months; y**= age in years, P***= Pearson Chi-Square or Fisher ‘s Exact test | | | | | | |
